# Factors Associated With Insomnia in Patients Undergoing Hemodialysis

**DOI:** 10.7759/cureus.22197

**Published:** 2022-02-14

**Authors:** Sofia Benetou, Victoria Alikari, Georgios Vasilopoulos, Maria Polikandrioti, Antonia Kalogianni, Georgios I Panoutsopoulos, Georgia Toulia, Dionyssios Leftheriotis, Georgia Gerogianni

**Affiliations:** 1 Department of Nursing, Postgraduate Program "Applied Clinical Nursing", University of West Attica, Athens, GRC; 2 Department of Nutritional Studies and Dietetics, University of Peloponnese, Kalamata, GRC; 3 Department of Cardiology, Attikon University Hospital of Athens, Athens, GRC

**Keywords:** depression, fatigue, insomnia, sleep disorders, hemodialysis

## Abstract

Introduction: Insomnia is the most common sleep disorder among patients on hemodialysis and has a strong relation with fatigue, depression, low immune system, increased risk of cardiovascular problems, and low quality of life. The aim of this study was to explore the factors associated with insomnia in patients undergoing hemodialysis.

Methods: In this cross-sectional study, 100 patients on hemodialysis (75 males and 25 females) from a hemodialysis center in Greece were included. Insomnia was assessed via the "Athens Insomnia Scale (AIS)" and a questionnaire about demographic and clinical characteristics. The Kruskal-Wallis, Mann-Whitney tests, and Spearman's rho criterion were used to evaluate the association between insomnia score and patients' characteristics. Multiple linear regression was performed to assess the effect of characteristics on patients' insomnia.

Results: Statistically significantly high levels of insomnia were found in patients over the age of 60 years (p = 0.002), in divorced/widowed patients (p = 0.007), in patients who had comorbid diseases (p = 0.001), in patients who felt tired after hemodialysis (p = 0.001), in those who had continuous fatigue (p = 0.001) and change in body image (p = 0.003), in those who often had itching (p = 0.007) and stiffness in joints (p = 0.001) and in patients who had limitations in the clothes they could wear (p = 0.001).

Conclusions: The findings of this study showed that insomnia had a strong association with increased age of patients, comorbidities, fatigue, change in body appearance, pruritus, and limitation in clothes they could wear. Therefore, there is a necessity for early assessment of sleep quality and effective treatment of sleep disorders in hemodialysis patients in order to reduce morbidity and mortality and improve the quality of their life.

## Introduction

Insomnia is the most common sleep disorder among patients on hemodialysis and its prevalence ranges from 69 to 80% [1]. Insomnia includes difficulty in initiating sleep and maintaining continuous sleep, awakening early in the morning or at night, and poor sleep quality [2,3]. These patients also have irregular sleeping hours, nightmares, morning headaches, and daytime sleepiness [4]. 

Hemodialysis patients are particularly concerned about insomnia since it has a strong relation with fatigue and low quality of life, while it usually leads to depression, low immune system, and increased risk of cardiovascular problems [2]. More than 50% of patients on hemodialysis have daytime sleepiness and fatigue, while more than 25% have depression [5]. Insomnia leads to a high risk of depression [3], while untreated depression can cause sleep disorders. More specifically, patients with sleep disturbances are three times more likely to have depressive disorders than those without sleep problems [4]. Additionally, daytime sleepiness is regarded as a significant factor for fatigue [6], poor concentration and irritability [3], decline in memory, and high levels of anxiety [7], while poor quality of sleep leads to low quality of life [4].

Insomnia is mostly affected by uraemic toxin accumulation, anemia, pain, and psychological problems [8]. It can be also caused by restless leg syndrome and itchy skin due to uremic pruritus [9], sleep apnoea [10], as well as by insufficient control of diabetes [11]. Diabetes usually causes sleep disturbances, while sleep problems may deteriorate the control of diabetes, such as diabetic retinopathy which is negatively affected by the irregular duration of sleep [11]. Additionally, sleep problems are usually common in hemodialysis patients with older age, as well as in those with a long time on hemodialysis and frequent hemodialysis sessions [3]. The long time on hemodialysis is possibly associated with an increasing appearance of symptoms and comorbidities, like muscle cramps, peripheral neuropathy, and bone pain which leads to poor sleep quality [4]. The aim of this study was to explore the factors associated with insomnia in patients undergoing hemodialysis.

## Materials and methods

Study sample

The study sample is a convenience sample consisting of 100 patients undergoing hemodialysis in a hemodialysis unit in Athens, Greece. Inclusion criteria were: age over 18 years old and less than 85 years old, on hemodialysis for at least three months, and ability to speak, read, and write in the Greek language. Exclusion criteria were insufficient language skills, age over 85 years old, cognitive impairment, and drug or alcohol abuse. All the participants were receiving regular hemodialysis three to four times per week. The study was carried out from November 2020 to January 2021.

Data collection was carried out by interviews using the questionnaire "Athens Insomnia Scale (AIS)" for the evaluation of insomnia and a questionnaire which was created by the researchers of this study and included: (a) socio-demographic characteristics like gender, age, marital status, number of children, educational level, occupation; (b) clinical characteristics: patients' comorbidities, the degree of information about their health problem, values of urea, creatinine, phosphorus, the level of stress they experienced due to their disease, if they wished to receive written instructions about the management of their disease, fatigue after hemodialysis and its duration, pain after hemodialysis, changes in body image, muscle cramps, stiffness, nausea, vomiting; (c) Information about patients' concerns: changes in social and personal life, changes in the amount of fluids and food they used to take, fear of the possibility of shutdown of the hemodialysis machine, difficulties in movement and clothing.

Athens Insomnia Scale (AIS)

The "Athens Insomnia Scale (AIS) is a self-assessment psychometric questionnaire designed to assess sleep difficulty based on the international diagnostic criteria ICD-10. It consists of eight questions, of which the first five refer to sleep initiation, awakenings during the night, final awakening, total sleep duration, and quality of sleep. The other three questions are related to well-being, functioning capability and tiredness during the day. The answers are scored on a scale of 0-3 and the total score of insomnia is evaluated by summing the scores, leading to a possible score ranging from 0 to 24. Higher scores indicate higher levels of insomnia. AIS has high consistency, reliability, and validity in Greek population and is a significant tool in clinical practice [12].

Statistical analysis

Nominal data are presented with absolute and relative (%) frequencies, while the continuous ones are presented with mean, standard deviation, median and interquartile range (IQR). The normality of the data was checked with the Kolmogorov-Smirnov criterion and graphically with histograms and Q-Q plots. The Kruskal-Wallis, Mann-Whitney tests and the spearman's rho criterion were used to evaluate the association between insomnia score and patients' characteristics. Multiple linear regression was performed to assess the effect of characteristics on patients' insomnia. The results are presented with β regression coefficients and 95% confidence intervals (CI). The observed significance level of 5% was considered statistically significant. All statistical analyzes were performed with version 25 of the SPSS program (IBM Corp., Armonk, NY).

Ethics

Before collecting data, we obtained approval from the Ethics Committee of General Hospital of Attica 'Sismanogleio- Amalia Fleming' (Νumber of approval:22/16-11-2020). Patients who met the inclusion criteria were informed during their scheduled treatment about the purpose and procedure of the study and the anonymity of the data. The study was carried out in accordance with the Declaration of Helsinki (1989).

## Results

A total of 100 patients participated in the study. Out of the total, 75% (n = 75) of them were male and 25% (n = 25) were female. The majority of the participants 73% (n=73) was over 60 years old and had two or more children (63%, n=63). Most of the respondents were married 65% (n=65) and pensioners 65% (n=65), while 42% (n=42) had primary education (Table 1).

**Table 1:** Demographic characteristics of participants (n=100) n = Number of participants

Patients' characteristics	n (%)
Gender	
Male	75 (75.0%)
Female	25(25.0%)
Age (years)	
30-40	2(2.0%)
41-50	9(9.0%)
51-60	16(16.0%)
61-70	30(30.0%)
>70	43(43.0%)
Status	
Single	14(14.0%)
Married	65(65.0%)
Divorced	3(3.0%)
Widowed	17(17.0%)
Living Together	1(1.0%)
Education Level	
Primary School	42(42.0%)
High School	46(46.0%)
University	10(10.0%)
MSc-PhD	2(2.0%)
Job	
Unemployed	4(4.0%)
Private Employee	3(3.0%)
Freelancer	16(16.0%)
Household	9(9.0%)
Pensioner	65(65.0%)
Other	3(3.0%)
Number of children	
0	14(14.0%)
1	23(23.0%)
2	45(45.0%)
>2	18(18.0%)

Additionally, 79% (n=79) were very or enough informed about their condition of health, 44% (n=44) suffered from another disease and 66% (n=66) had very or enough anxiety about the progression of the disease, 36% (n=36) believed that regular updating was very helpful in reducing stress, 64% (n=64) wanted to receive written information on disease management, 16% (n=16) weighed themselves daily and 15% (n=15) stated that they had insomnia. Moreover, 34% (n=34) felt tired after the hemodialysis session, 39% (n=39) felt more tired at night and 84% (n=84) felt tired for a few hours, 37% (n=37) sometimes felt pain during venus-puncture, 73% (n=73) believed they had change in body image and 34% (n=34) had itching sometimes. Frequent muscle cramps had 11% (n=11) and stiffness 10% (n=10), while 14% (n=14) had nausea and vomiting sometimes. The mean values of the patients' recent urea, creatinine, and phosphorus were 124, 9, and 6.2, respectively.

Moreover, 65% (n=65) were bothered by spending a lot of time on dialysis, 55% (n=55) were concerned that they had limited social life due to hemodialysis, and 69% (n=69) stated that they had changes in their role as a husband/wife. Additionally, 27% (n=27) had a restriction on the clothes they could wear, 31% (n=31) wanted to hide a part of their body and 70% (n=70) said that they had experienced a change in their body image (Table 2).

**Table 2:** Patients' concerns (n=100) n = Number of participants

Patients' concerns	n (%)
Does the fact that you spend a lot of time on dialysis bother you?	
Yes	65(65.0%)
No	10(10.0%)
Sometimes	25(25.0%)
Are you concerned about the fact that you now have a limited social life compared to what you had before you started dialysis?	
Yes	55(55.0%)
No	13(13.0%)
Sometimes	27(27.0%)
Often	5(5.0%)
Are there any changes in your role as a husband/wife?	
Yes	69(69.0%)
No	19(19.0%)
Sometimes	10(10.0%)
Often	2(2.0%)
Are you concerned about the fact that you have to take a limited amount of fluids?	
Yes	74(74.0%)
No	5(5.0%)
Sometimes	17(17.0%)
Often	4(4.0%)
Are you concerned about the fact that you have to avoid certain foods that you crave?	
Yes	65(65.0%)
No	10(10.0%)
Sometimes	22(22.0%)
Often	3(3.0%)
Are you concerned about the possibility of disruption of arteriovenous anastomosis?	
Yes	56(56.0%)
No	8(8.0%)
Sometimes	34(34.0%)
Often	2(2.0%)
Are you worried about the possibility of the dialysis machine shutting down?	
Yes	16(16.0%)
No	47(47.0%)
Sometimes	37(37.0%)
Are you worried about your frequent travel to and from the Dialysis Unit?	
Yes	59(59.0%)
No	16(16.0%)
Sometimes	21(21.0%)
Often	4(4.0%)
Do you have trouble going on vacation?	
Yes	86(86.0%)
No	4(4.0%)
Sometimes	9(9.0%)
Often	1(1.0%)
Do you have any restrictions on the clothes you can wear?	
Yes	27(27.0%)
No	36(36.0%)
Sometimes	36(36.0%)
Often	1(1.0%)
Do you want to hide or conceal your body or any part of your body? (like the fistula)	
Yes	31(31.0%)
No	47(47.0%)
Sometimes	22(22.0%)
Do you experience a change in body image?	
Yes	70(70.0%)
No	26(26.0%)
Sometimes	4(4.0%)

Regarding the scoring on AIS, the median value was 6 (interquartile range: 4-9.5) as at least 50% of patients scored below 6 while the mean score was 7.1 (SD: 4.9). In addition, 25% of patients scored below 4. These values with respect to the possible range of score (0-24) indicate low levels of patient insomnia.

Association of insomnia scale with patients' characteristics

Statistically significantly high levels of insomnia were found in patients over the age of 60 years old (p =0.002), in divorced/widowed patients (p = 0.007), in patients who had comorbid diseases (p = 0.001), in patients who felt tired after hemodialysis (p = 0.001), in those who had continuous fatigue (p = 0.001) and change in body image (p = 0.003), in those who often had itching (p = 0.007) and stiffness in joints (p = 0.001) and in patients who had limitations in the clothes they could wear (p = 0.001).

More specifically, patients over the age of 60 years old had statistically significantly higher levels of insomnia (median 7) than younger patients (median 3). Divorced/widowed patients had statistically significantly higher levels of insomnia (median 9) than married and single patients (median 5). Patients who had another disease had higher levels of insomnia (median 9) than those who did not have another disease (median 4). In addition, higher levels of insomnia were experienced by those who felt tired after hemodialysis (median 10), those who had continuous fatigue (median 11), those who felt that they had a change in body image (median 7), those who often felt itching and stiffness (median 12 and 10 respectively), and those who had limitations in the clothes they could wear (median 7) (Table 3).

**Table 3:** Association of insomnia scale with patients' characteristics

Patients' characteristics	Mean (SD)	Median (IQR)	p-value
Gender			0.971
Male	7.2(5.2)	6(4-10)	
Female	6.8(4.2)	6(4-9)	
Age (years)			0.002
≤60	4.7(4.3)	3(2-7)	
61-70	7.2(4.5)	7(4-10)	
>70	8.4(5.1)	7(5-11)	
Status			0.007
Married / Living Together	6.4(4.6)	5(3-9)	
Single	6.1(5.2)	5(2-11)	
Divorced / Widowed	10.0(4.9)	9(6-12)	
Education Level			0.092
Primary School	8.2(5.0)	7(4-12)	
High School	6.3(5.1)	5(3-9)	
University/ MSc-PhD	5.8(3.5)	5(3-9)	
Job			0.347
Unemployed/ Household	6.5(4.1)	6(4-8)	
Employee	6.1(4.9)	5(3-9)	
Pensioner	7.6(5.1)	7(4-10)	
Number of children			0.162
0	5.4(4.6)	4(2-9)	
1	8.0(4.5)	9(6-10)	
2	7.5(5.3)	5(4-9)	
>2	6.1(4.5)	5(3-8)	
Informed about the health problem			0.533
Very	5.6(4.9)	5(1-9)	
Enough	7.3(5.0)	6(4-10)	
A little / Not at all	7.1(4.8)	6(5-8)	
Other diseases			0.001
Yes	9.7(5.0)	9(6-12)	
No	5.0(3.7)	4(3-7)	
Are you nervous about the progression of the disease?			0.643
Very	7.4(4.4)	6(4-11)	
Enough	6.5(4.1)	6(4-9)	
A little / Not at all	7.4(6.1)	6(3-9)	
Do you believe that regular updating helps reducing stress?			0.149
Very	7.4(4.5)	7(4-11)	
Enough	5.8(4.3)	5(3-7)	
A little / Not at all	8.5(6.0)	7(5-12)	
Do you wish to receive written information regarding the management of the disease?			0.100
Yes/Sometimes	6.5(4.5)	6(4-9)	
No	8.8(5.8)	7(4-12)	
How often do you weigh yourself at home?			0.072
Daily	4.6(3.4)	4(3-7)	
Every 2-4 days	6.0(4.8)	7(0-10)	
Once per week	7.7(5.1)	6(4-10)	
Do you feel tired after each dialysis session?			0.001
Yes	10.2(5.6)	10(6-14)	
Sometimes / Often	5.6(3.7)	5(3-8)	
When do you feel most tired?			0.083
Morning / Noon	8.8(5.1)	9(5-11)	
Afternoon	6.1(4.4)	5(4-7)	
Night	6.8(5.1)	6(3-9)	
What is the duration of fatigue?			0.001
Continuous	13.2(5.6)	11(10-18)	
Some hours	6.0(4.0)	5(3-8)	
Do you feel pain during venipuncture?			0.761
No	6.5(5.0)	5(3-10)	
Rarely	6.8(2.8)	7(4-9)	
Sometimes	7.3(5.1)	6(4-9)	
Often / Yes	7.9(6.9)	6(2-15)	
Do you think there is a change in body image after the diagnosis of the disease?			0.003
Yes	7.8(5.0)	7(4-11)	
No	5.0(3.9)	4(2-7)	
Have you had itching in your body since you started hemodialysis?			0.007
No	5.9(3.9)	5(3-10)	
Rarely/Sometimes	6.8(5.1)	6(4-9)	
Often / Yes	10.8(5.2)	12(6-15)	
Do you have muscle cramps?			0.228
No	6.9(3.8)	6(4-10)	
Rarely / Sometimes	6.7(5.2)	5(3-9)	
Yes / Often	8.9(5.4)	8(5-12)	
Do you have stiffness in your joints?			0.001
No	5.0(3.7)	4(3-7)	
Rarely / Sometimes	6.7(5.4)	5(4-9)	
Yes / Often	10.3(4.0)	10(7-12)	
Do you feel nausea and vomiting?			0.843
No	6.6(4.0)	6(4-9)	
Rarely / Sometimes	7.5(6.1)	6(4-12)	
Are you concerned about the fact that you have a limited social life compared to what you had before you started dialysis?			0.575
Yes/ Sometimes	7.0(4.5)	6(4-9)	
No	7.5(7.4)	6(2-11)	
Are there any changes in your role as a husband/wife?			0.561
Yes/ Sometimes	6.7(4.2)	6(4-9)	
No	8.5(7.2)	7(2-12)	
Are you worried about the possibility of the dialysis Machine shutting down?			0.297
Yes/ Sometimes	6.6(4.6)	5(3-9)	
No	7.6(5.2)	7(4-10)	
Do you have any restrictions on the clothes you can wear?			0.001
Yes/ Sometimes	7.5(4.3)	7(4-10)	
No	6.2(5.8)	5(3-7)	
Do you want to hide or conceal your body or any part of your body? (like the fistula)			0.701
Yes/ Sometimes	7.0(4.4)	6(4-10)	
No	7.2(5.5)	6(3-9)	
	Spearman’s Rho	p-value	
Recent Urea	-0.079	0.435	
Recent Creatinine	-0.180	0.074	
Recent Phosporus	-0.118	0.244	
IQR: Interquartile range, SD: Standard Deviation			

Effect of patients' characteristics on insomnia scale

Multiple linear regression was performed to assess the effect of patient characteristics (independent factors) on the insomnia scale. We observe that divorced/widowed patients had 2 points higher insomnia score than married patients (β = 2.15, 95% CI: -0.02-4.32, p = 0.052). Patients who sometimes felt tired after dialysis had a statistically significantly lower insomnia score of 2.2 points than patients who felt tired each time after dialysis (β = -2.21, 95% CI: -4.40--0.01, p = 0.049). In addition, patients who had fatigue for a few hours had a statistically significantly lower insomnia score of 3.6 points than those who had continuous fatigue (β = -3.60, 95% DE: -6.69-0.51, p = 0.023) (Table 4).

**Table 4:** Effect of patients' characteristics on insomnia scale

Patients' characteristics	β coefficient (95% CI)	p-value
Age (years)		
≤60	Ref. Cat.	
61-70	0.73(-1.62-3.07)	0.538
>70	1.71(-0.61-4.04)	0.147
Status		
Married / Living Together	Ref. Cat.	
Single	0.10(-2.68-2.87)	0.945
Divorced / Widowed	2.15(-0.02-4.32)	0.052
Other disease		
Yes	Ref. Cat.	
No	-1.21(-3.25-0.82)	0.240
Do you feel tired after each dialysis session?		
Yes	Ref. Cat.	
Sometimes / Often	-2.21(-4.40--0.01)	0.049
What is the duration of fatigue?		
Continuous	Ref. Cat.	
Some hours	-3.60(-6.69--0.51)	0.023
Do you think there is a change in body image after the diagnosis of the disease		
Yes	Ref. Cat.	
No	-0.70(-3.35-1.96)	0.603
Have you had itching in your body since you started dialysis?		
No	Ref. Cat.	
Rarely/Sometimes	0.36(-1.79-2.52)	0.737
Often / Yes	2.04(-0.93-5.02)	0.176
Do you have stiffness in your joints?		
No	Ref. Cat.	
Rarely / Sometimes	-0.85(-3.51-1.81)	0.526
Yes / Often	0.24(-3.14-3.62)	0.887
CI: Confidence Interval		

## Discussion

The present study found that patients over 60 years old had statistically significantly higher levels of insomnia than younger patients. Similarly, in a previous study, it was found that a frequent complaint among elderly patients on hemodialysis was the early awakening in the morning [13]. It can be assumed that sleep problems in elderly people on hemodialysis are mostly caused by depressive disorders, cardiovascular problems, systemic inflammation, and comorbid diseases [14] leading to physical, cognitive, and emotional deterioration [15].

Moreover, this study showed that divorced/widowed patients had statistically significantly higher levels of insomnia than married and single patients. It can be attributed to the fact that patients undergoing hemodialysis face problems with marital adjustment since this treatment increases stress, anxiety, and depression due to changes in marital roles [16].

The findings of this study indicated that individuals with other diseases had higher levels of insomnia than those who did not have any other diseases. Insomnia frequently appears in people with comorbid diseases [17]. This can be viewed in the context of general health status since comorbid diseases include a variety of complications, disabilities, and chronic pain. Chronic pain is related to frequent hospitalizations, low quality of life, increased rates of mortality, and increased stress. Musculoskeletal is the most troublesome pain, which frequently leads to sleep problems [4]. Similarly, Hamzi et al. found that hemodialysis patients who required parathyroidectomy were sleeping a few hours and had a high prevalence of sleep problems [18]. It is important to take into consideration that increased levels of parathyroid hormone are related to renal bone disease and pain in bones [17].

The present study also found that patients who felt tired after dialysis and those who had continuous fatigue had statistically significantly higher levels of insomnia. These findings are congruent with those of a previous study indicating that there was a significant relationship between fatigue and self-reported quality of sleep, excessive daytime sleepiness, and restless leg symptoms [6]. This can be attributed to the fact that sleep disturbances lead to excessive daytime sleepiness and high levels of inflammatory cytokines which cause fatigue in dialysis patients [6]. In a similar study, it was found that sleep disorders and fatigue were more frequent in hemodialysis patients with restless leg syndrome [19]. It can be assumed that restless leg syndrome leads to low quality of sleep since patients have the need to move their legs or other parts of their body during their sleep [4].

The findings of the present study indicated that patients who often felt itching experienced higher levels of insomnia. Pruritus in chronic kidney disease is a common and irritating complication that leads to sleep disorders and low quality of life [20]. Soleymanian et al. found that pruritus was a significant predictor for insomnia [21]. Similarly, Orasan et al. found that survival at 20 months was lower in patients with both pruritus and insomnia [22], while in another study low quality of sleep was found to be related to increased mortality in hemodialysis patients [23]. It can be assumed that uremic pruritus has a negative effect on the general health functioning of hemodialysis patients since it is associated with systemic inflammation and cardiovascular mortality [24].

Moreover, the findings of this study showed that patients who felt that they had a change in body image and those who felt a limited choice in the clothes they could wear experienced high levels of insomnia. The body appearance of hemodialysis patients is negatively affected by the creation of arterio‐venous fistula, the repeated use of central venous catheters, generalized edema, weight loss, and several surgical scars. It can be assumed that changes in body appearance lead to limitations in the clothes they can wear, which has been experienced as a serious stressor among these people [25].

The results of this study offer significant information to health professionals about the factors associated with insomnia in patients undergoing hemodialysis. The findings of this study showed that insomnia had a strong association with increased age of patients, comorbidities, fatigue, change in body appearance, pruritus, and limitation in clothes they could wear. Therefore, this study raises the issue of the necessity for early assessment of sleep quality and effective treatment of sleep disorders in patients on hemodialysis in order to reduce morbidity and mortality and improve the quality of their life.

Limitations

This study has some limitations. Convenience sampling is one of the limitations since the study was conducted only in one hemodialysis unit in Athens. Thus the sample is not representative of all patients undergoing hemodialysis in Greece and the findings cannot be generalized. Secondly, there was no other assessment to evaluate insomnia in hemodialysis patients in this study.

## Conclusions

Insomnia is a common problem among hemodialysis patients leading to poor quality of life. The results of this study showed higher levels of insomnia in patients over 60 years old, patients with other diseases, those who experienced fatigue after hemodialysis, those who thought they had a change in body image, those who often felt itchy and felt restricted in the clothes they could wear. The hemodialysis population is of an increased age with high levels of insomnia. Thus, insomnia and other sleep disorders should be early assessed and effectively treated by health professionals in order to improve the quality of sleep in these people.

## Appendices

Athens Insomnia Scale 

This scale (Table 5) is intended to record your own assessment of any sleep difficulty you might have experienced. Please, check (by circling the appropriate number) the items below to indicate your estimate of any difficulty, provided that it occurred at least three times per week during the last month.

**Table 5:** Athens Insomnia Scale

1. SLEEP INDUCTION ( the time it takes you to fall asleep after turning off the lights)			
0 No problem	1 Slightly delayed	2 Markedly delayed	3 Very delayed or did not sleep at all
2. AWAKENINGS DURING THE NIGHT			
0 No problem	1 Minor problem	2 Considerable problem	3 Serious problems or did not sleep at all
3. FINAL AWAKENING EARLIER THAN DESIRED			
0 Not earlier	1 A little earlier	2 Markedly earlier	3 Much earlier or did not sleep at all
4. TOTAL SLEEP DURATION			
0 Sufficient	1 Slightly insufficient	2 Markedly insufficient	3 Very insufficient or did not sleep at all
5. OVERALL QUALITY OF SLEEP (no matter how long you slept)			
0 Satisfactory	1 Slightly unsatisfactory	2 Markedly unsatisfactory	3 Very unsatisfactory or did not sleep at all
6. SENSE OF WELL-BEING DURING THE DAY			
0 Normal	1 Slightly decreased	2 Markedly decreased	3 Very decreased
7. FUNCTIONING (PHYSICAL AND MENTAL) DURING THE DAY			
0 Normal	1 Slightly decreased	2 Markedly decreased	3 Very decreased
8. SLEEPINESS DURING THE DAY			
0 None	1 Mild	2 Considerable	3 Intense
